# Leaf area estimation in small-seeded broccoli using a lightweight instance segmentation framework based on improved YOLOv11-AreaNet

**DOI:** 10.3389/fpls.2025.1622713

**Published:** 2025-07-04

**Authors:** Yaben Zhang, Yifan Li, Xiaowei Cao, Zikun Wang, Jiachi Chen, Yingyue Li, Zhibo Zhong, Ruxiao Bai, Peng Yang, Feng Pan, Xiuqing Fu

**Affiliations:** ^1^ College of Engineering, Nanjing Agricultural University, Nanjing, China; ^2^ Institute of Farmland Water Conservancy and Soil-Fertilizer, Xinjiang Academy of Agricultural Reclamation Science, Shihezi, Xinjiang, China; ^3^ Institute of Mechanical Equipment, Xinjiang Academy of Agricultural Reclamation Science, Shihezi, Xinjiang, China

**Keywords:** broccoli seedlings, improved YOLOv11, lightweight model, leaf area segmentation, plant trait quantification, smart agriculture

## Abstract

**Introduction:**

Accurate leaf area quantification is vital for early phenotyping in small-seeded crops such as broccoli (Brassica oleracea var. italica), where dense, overlapping, and irregular foliage makes traditional measurement methods inefficient.

**Methods:**

This study presents YOLOv11-AreaNet, a lightweight instance segmentation model specifically designed for precise leaf area estimation in small-seeded broccoli seedlings. The model incorporates an EfficientNetV2 backbone, Focal Modulation, C2PSA-iRMB attention, LDConv, and CCFM modules, optimizing spatial sensitivity, multiscale fusion, and computational efficiency. A total of 6,192 germination-stage images were captured using a custom phenotyping system, from which 2,000 were selected and augmented to form a 5,000-image training set. Post-processing techniques—including morphological optimization, edge enhancement, and watershed segmentation—were employed to refine leaf boundaries and compute geometric area.

**Results:**

Compared to the original YOLOv11 model, YOLOv11-AreaNet achieves comparable segmentation accuracy while significantly reducing the number of parameters by 57.4% (from 2.84M to 1.21M), floating point operations by 25.9% (from 10.4G to 7.7G), and model weight size by 51.7% (from 6.0MB to 2.9MB), enabling real-time deployment on edge devices. Quantitative validation against manual measurements showed high correlation (R² = 0.983), confirming the system’s precision. Additionally, dynamic tracking revealed individual growth differences, with relative leaf area growth rates reaching up to 26.6% during early germination.

**Discussion:**

YOLOv11-AreaNet offers a robust and scalable solution for automated leaf area measurement in small-seeded crops, supporting high-throughput screening and intelligent crop monitoring under real-world agricultural conditions.

## 1 Introduction

With the accelerating shift toward digitalization and intelligent systems in agriculture, the accurate and efficient extraction of crucial phenotypic characteristics has emerged as a central issue in plant science and precision farming. Notably, leaf area represents a major physiological parameter, directly impacting processes like photosynthesis, transpiration, biomass development, and final crop yield (Richards, 2000; Kruger and Volin, 2006; Santesteban and Royo, 2006).In research areas including genetic improvement, targeted cultivation, and ecological adaptability studies, technologies for automating leaf area assessment have shown substantial practical relevance. As the demand for high-throughput phenotyping intensifies, traditional manual or semi-automatic approaches often fall short in meeting the speed and precision expectations of both academic and real-world agricultural applications.

Broccoli (*Brassica oleracea* var. *italica*), a widely cultivated and nutritionally dense cruciferous crop, is recognized for its abundant health-promoting compounds and bioactive properties, including anti-cancer, antioxidant, and anti-inflammatory effects (Lugasi et al., 1999; Yong and Kyung, 2021). As molecular breeding and rapid selection technologies advance, broccoli germplasm studies have transitioned into stages characterized by high-precision and high-throughput phenotypic analysis. The research emphasis has shifted from basic trait evaluation to early-stage quantification of detailed phenotypic traits. Within this context, reliably and efficiently extracting leaf area during the initial seedling phase has become a major bottleneck, constraining both the throughput of germplasm screening and the quality of breeding decisions.Broccoli seeds are classified as small-seeded types, and during the seedling stage, the leaves tend to exhibit characteristics such as small size, high density, diverse shapes, and frequent occlusion or overlap, which greatly increases the difficulty of automatic leaf area measurement (Heather and Sieczka, 1991). In practical applications, traditional methods relying on manual measurement or leaf scanning suffer from significant limitations in terms of efficiency, subjective error, and operational complexity—especially when handling large-scale, multi-temporal datasets (Kusuda, 1994). Therefore, the development of a leaf area extraction technology suitable for small-seeded plants that provides high precision, strong robustness, and automation capability has become a key element in achieving agricultural intelligence and high-throughput phenotyping.

In recent years, deep learning, especially convolutional neural networks (CNNs), has shown great potential in the field of agricultural image processing. YOLO (You Only Look Once)-series models, known for their efficient end-to-end detection and real-time performance, have been widely applied in tasks such as pest and disease identification, fruit counting, and weed detection (Deng et al., 2021; Washburn et al., 2021). For example, Deng et al. proposed combining Faster R-CNN with Feature Pyramid Networks (FPN) to automatically count rice spikelets, achieving 99.4% accuracy even under complex background (Deng et al., 2021); Castro-Valdecantos et al. trained deep models using RGB images to estimate maize leaf area index, significantly improving the accuracy of agricultural remote sensing analysic (Castro-Valdecantos et al., 2022); Hamila et al. leveraged multispectral point clouds and 3D convolutional networks to spatially detect and assess the severity of Fusarium head blight in wheat (Hamila et al., 2024); and Masuda et al. combined CNNs with transcriptomic data to identify and predict key physiological changes in persimmon fruit softening (Masuda et al., 2023).While YOLO-based object detection frameworks are widely adopted in agricultural imaging, their standard outputs—bounding boxes and classification tags—are inadequate for pixel-level segmentation of intricate, flexible, and overlapping structures like plant foliage. This shortfall becomes especially problematic when dealing with small, densely clustered elements such as broccoli seedling leaves, where accurately tracing leaf boundaries is vital for precise area estimation. Further complicating these tasks are environmental variables including changing illumination, complex backgrounds, and frequent occlusions between adjacent leaves.

In efforts to enhance segmentation accuracy for visually complex targets, instance segmentation has gained increasing traction as a promising solution. By integrating object detection with semantic segmentation, this technique facilitates both classification and pixel-level mask generation, offering deeper interpretability in agricultural images (Yang et al., 2024). In recent developments, YOLO architectures have been adapted to perform instance segmentation, as seen in models like YOLOv5-seg and YOLOv8-seg, which have found growing use in detecting leaves, fruits, and disease regions. For instance, Kumar et al. incorporated a Bi-FAPN module using YOLOv5 and DenseNet-201 for early-stage rice disease recognition (Kumar et al., 2023); Sampurno et al. implemented YOLOv8n-seg on robotic weeders, yielding over 76% precision in natural field conditions (Sampurno et al., 2024); Yuan et al. combined YOLOv8 with drone-captured multispectral data to segment Chinese cabbage seedlings with a mAP of 86.3% (Yuan et al., 2024); and Khan et al. enhanced YOLOv8 with dilated convolution and GELU activations to achieve 93.3% accuracy in orchard canopy segmentation (Khan et al., 2024).Despite recent advances, many current instance segmentation models continue to struggle with small, irregularly shaped targets. Challenges such as excessive architectural complexity, sluggish inference, and imprecise boundary extraction persist (Chen et al., 2021). These limitations are especially pronounced for crops like broccoli, where overlapping and densely packed seedling leaves demand advanced edge and contour learning. Additionally, the heavy parameter loads in many existing models impede their deployment on edge devices or in real-time field applications (Liu et al., 2025), where efficiency and responsiveness are critical.

To address the above limitations, we developed YOLOv11-AreaNet for segmenting broccoli seedling leaves. It uses EfficientNetV2 as the backbone, with reduced width and depth (0.25 and 0.5) to improve efficiency.A Focal Modulation layer is embedded in the sixth stage to improve contextual awareness and sensitivity to local features. For finer recognition of small structures, we introduce the lightweight attention mechanism C2PSA_iRMB to sharpen feature focus while preserving speed. Additionally, the network’s original PANet and ASF modules are replaced by CCFM (Context-aware Cross-scale Fusion Module), which adaptively fuses multi-resolution features using a gated, multi-branch configuration—striking a balance between semantic abstraction and detail resolution. LDConv, a lightweight deformable convolution, substitutes standard layers to further cut computational load. Together, these optimizations enable accurate, efficient, and robust instance segmentation in multi-target, multi-time broccoli phenotyping tasks.

This research builds upon a custom-designed phenotyping platform developed for seed germination studies, used to collect and annotate broccoli seedling images across various growth stages. The YOLOv11-AreaNet framework was then applied for high-resolution instance segmentation, followed by downstream processing and time-series leaf area analysis. Comparative experiments revealed that our model significantly outperforms classical YOLO variants and established segmentation methods in terms of detection accuracy for small objects, boundary delineation, segmentation robustness, and inference latency. The system supports autonomous monitoring of small-seeded plant development under natural environments, providing valuable tools for early-stage breeding, seedling health diagnostics, and precision agricultural interventions. Additionally, this approach holds potential for application in other small-seeded crop species with similar phenotyping challenges.

## 2 Materials and methods

### 2.1 Experiment equipment

**Figure 1:**
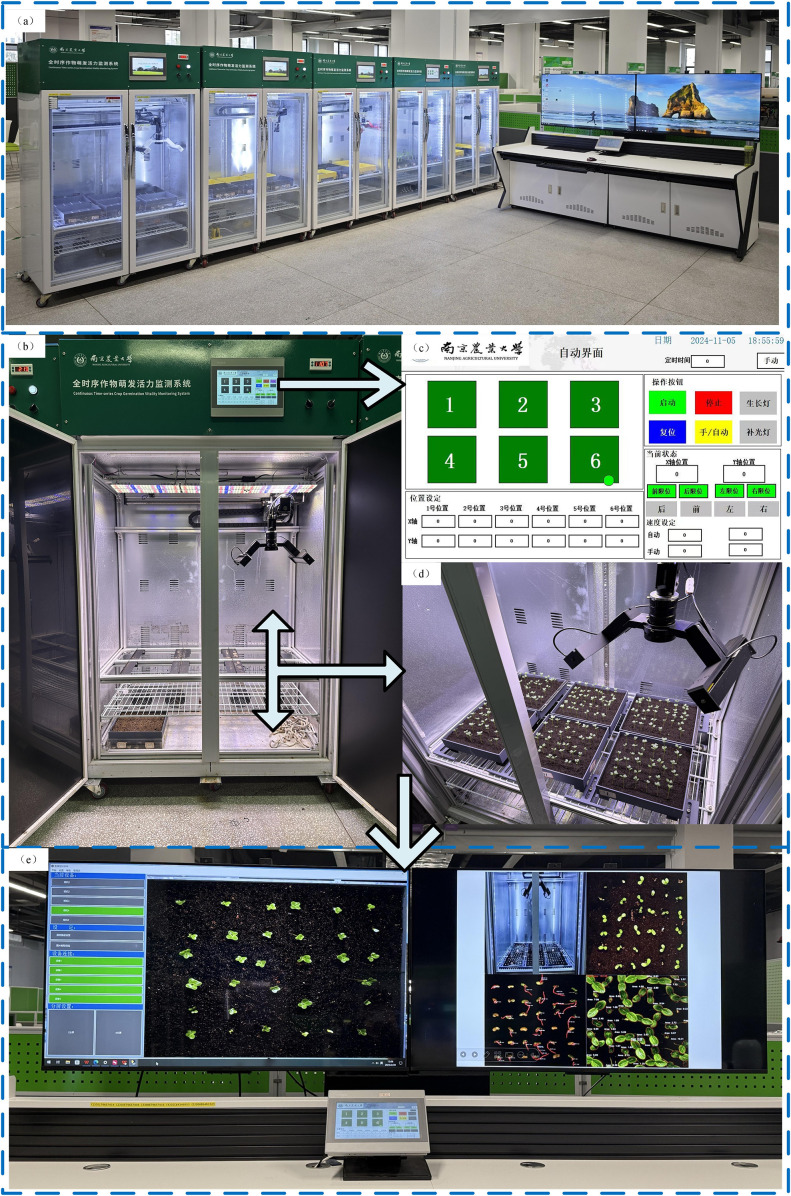
**(a)** Overall view of the full time-series crop growth monitoring system. **(b)** Continuous Time-series Crop Growth Vitality Monitoring System. **(c)** human-computer interaction interface. **(d)** image acquisition setup. **(e)** real-time monitoring software.

A full-time-sequence monitoring platform for crop growth vitality was employed in this study, as illustrated in **Figure 1**. The system integrates a seed germination chamber, an industrial image capture module, a human–machine interaction interface, and real-time status monitoring components, facilitating continuous observation of plant phenotypic traits. The operational workflow is detailed in **Figure 1**.

The germination chamber is equipped with independently adjustable temperature and lighting controls, supporting experimental conditions ranging from 5°C to 50°C. Illumination is uniformly provided through a dedicated LED array. Multiple custom-made culture trays (25 cm × 25 cm) can be placed simultaneously within the chamber to enable parallel observation of different experimental groups. These trays are fabricated using 3D printing technology, specifically designed to minimize acrylic surface reflection.The image capture component includes a HIK Vision industrial camera mounted on a horizontal rail, driven by a stepper motor for precise linear motion. High-resolution images (2592 × 2048 pixels) are captured dynamically and transmitted via GigE to the host system. Users can fine-tune focal length, capture intervals, and pre-cropping parameters through the software interface. The PLC module automatically stores the acquired images in a designated directory, where they undergo preprocessing for dataset generation.This configuration supports uninterrupted time-series image collection of the germination process, establishing a robust foundation for automated analysis of seedling phenotype dynamics using instance segmentation techniques.

### 2.2 Data collection and preprocessing

#### 2.2.1 Data collection

**Figure 2:**
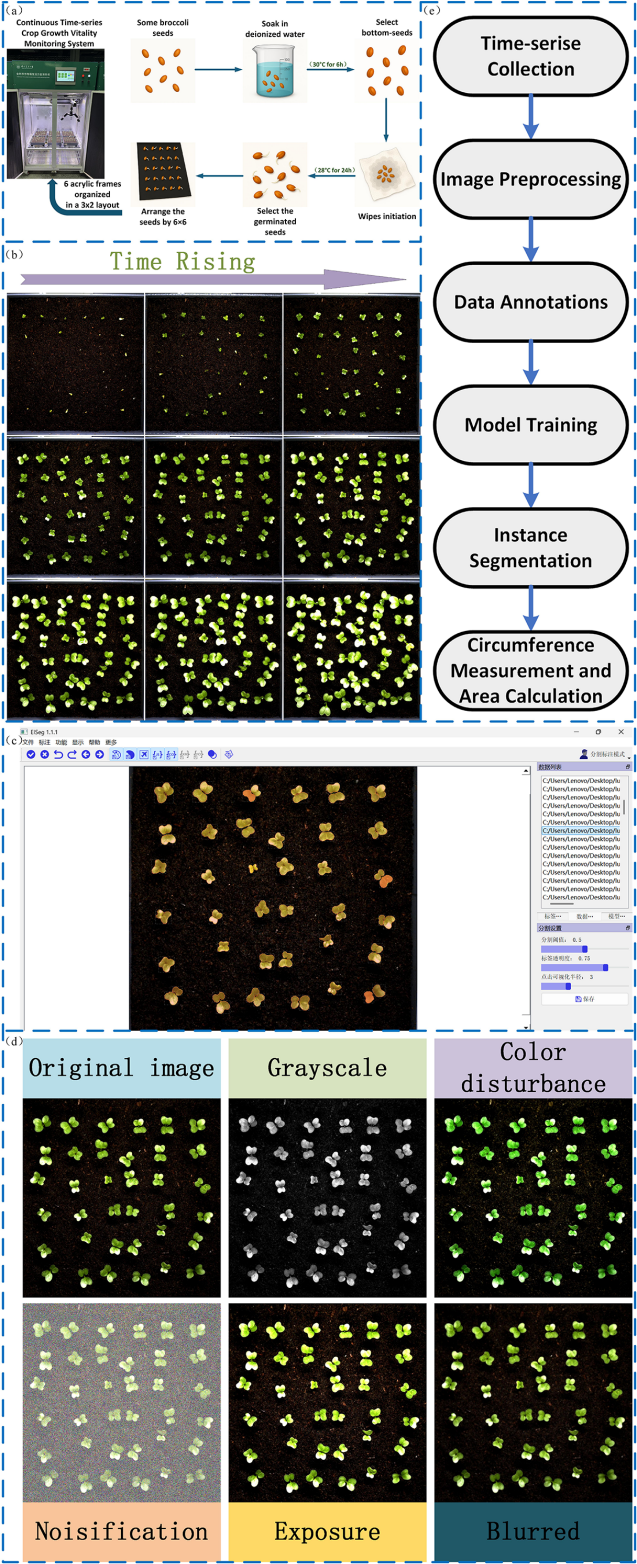
**(a)** Seed preparation process, including soaking, selection, and arrangement. **(b)** Time-series images showing seedling growth progression. **(c)** Image labeling of seeds. **(d)** Data augmentation techniques applied to seed images. **(e)** System workflow for time-series data collection, model training, and area calculation.

To construct a growth rate model for leaf area in broccoli seedlings, we selected 300 broccoli seeds with uniform size and intact morphology, strictly screened according to appearance and size standards to ensure data reliability and reproducibility. The seeds were soaked in deionized water at 30°C for 6 hours to activate cellular growth mechanisms and ensure optimal hydration. After soaking, seeds that had settled to the bottom of the container were collected and evenly laid on a moist towel, placed inside a germination chamber, and subjected to 24 hours of priming treatment in an incubator with constant temperature (28°C) and continuous illumination to ensure optimal germination conditions.Following priming, 216 healthy and full seeds were selected and arranged in a 6×6 pattern in each culture box. The culture boxes were then placed in a 3×2 configuration inside a constant temperature and constant light incubator, as shown in **Figure 2a**, and seedling monitoring began for a period of 11 days. The experimental environment parameters (temperature, humidity, illumination) were strictly controlled to ensure growth stability. Specific experimental parameters are listed in **Table 1**.

**Table 1:** Experiment parameters.

Number of seeds per plate	36
Incubation temperature	28°C
Shooting interval	15min
Total number of images	6192
Image cropping resolution	1500×1500
Picture format	JPG
The ratio of training, validation and prediction	7:2:1

During the experiment, the plant seed germination phenotyping system captured images every 15 minutes, recording leaf area changes from germination to seedling stage for growth model construction.

#### 2.2.2 Data pre-processing

A total of 6,192 germination images were collected using the plant seed germination phenotyping system. Since germination phenomena were not obvious during the first 48 hours and leaves had not yet emerged, images from this time period were excluded. Subsequently, 2,000 images were selected from the remaining dataset to construct the dataset for model training. **Figure 2b** illustrates the germination and growth process of the broccoli seedlings. Image annotation was performed using the eiseg software, as shown in **Figure 2c**, treating all leaves as a single category labeled as “SEED.” After annotation, We applied a series of data augmentation techniques to enhance leaf edge clarity, improve recognition under complex backgrounds, and reduce overfitting, as shown in **Figure 2d**. These augmentation techniques were not only applied to increase the diversity of the dataset but were also carefully selected to simulate typical visual disturbances encountered in real-world agricultural environments. Specifically, exposure adjustments emulate challenges caused by strong backlighting or localized shadows; grayscale conversion and color perturbations mimic color distortions and reduced saturation resulting from reflective mulch, soil backgrounds, or uneven natural illumination; and noise or blur effects correspond to sensor noise, motion blur, or defocus that frequently occur during high-throughput field image acquisition. Incorporating these perturbations during training helps the model learn more robust feature representations and enhances its generalization capability under practical deployment scenarios. Ultimately, we obtained 5,000 images, which were divided into training, validation, and test sets at a ratio of 7:2:1. **Figure 2e** illustrates the overall workflow of this study, including time-series data collection, image preprocessing, annotation, model training, instance segmentation, and leaf area calculation.

### 2.3 YOLOv11 model optimization

With the great success achieved by YOLOv11 in the field of computer vision, especially in object detection, it has become one of the most accurate and fastest detection models currently available (Jiang et al., 2022). However, in practical agricultural applications, many devices are limited by computational capacity, requiring the reduction of model complexity through optimization while maintaining high accuracy, so that it can be deployed on embedded systems or mobile platforms. Therefore, we conducted multiple optimizations based on YOLOv11 to ensure the retention of accuracy while successfully achieving model lightweighting.

**Figure 3:**
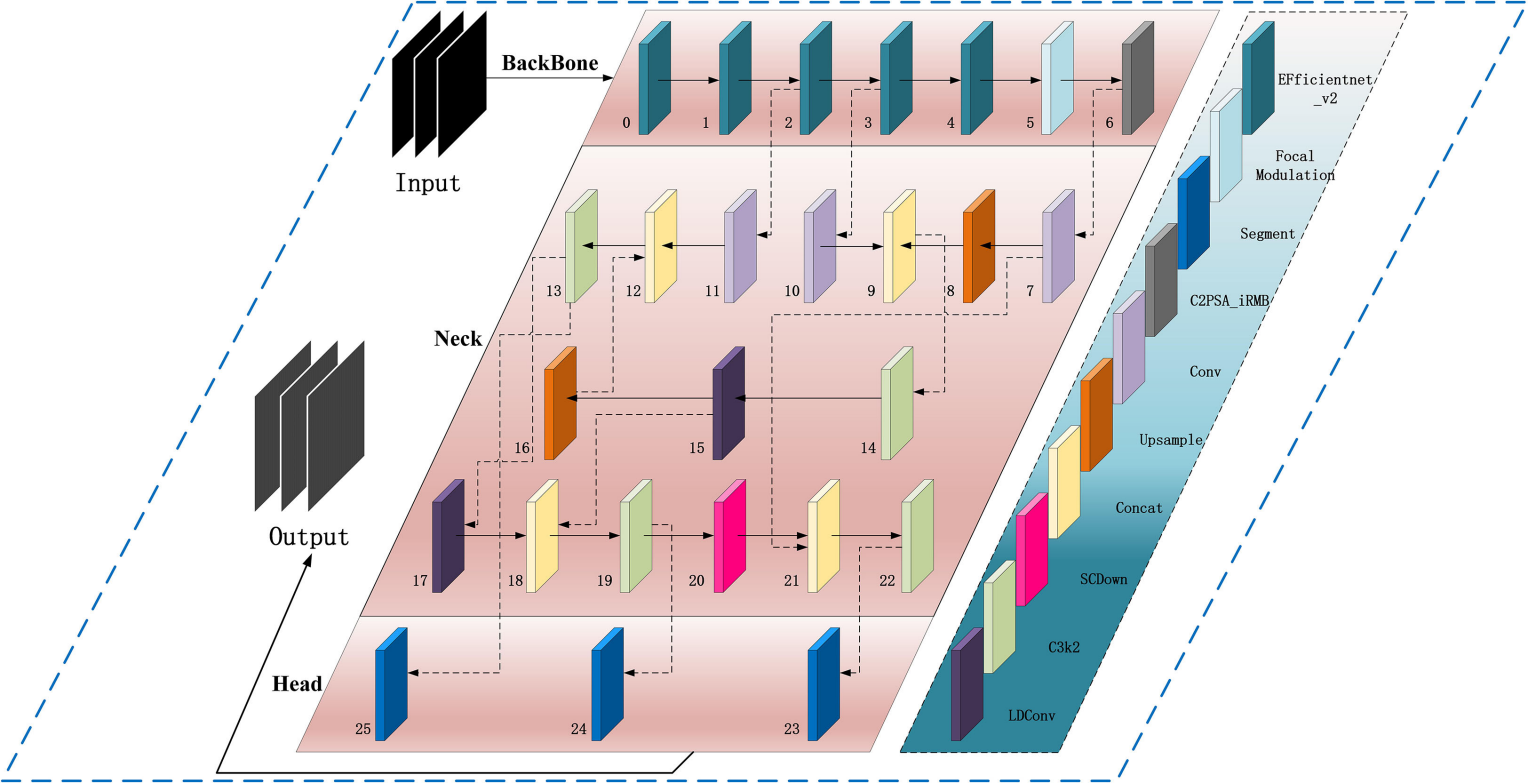
Improved YOLOv11 segmentation model architecture.

We propose an improved YOLOv11-AreaNet model for leaf area segmentation, based on YOLOv11 with added new modules.The main improvements include the EfficientNetV2 backbone network, Focal Modulation, C2PSA-iRMB, LDConv, and CCFM modules. The introduction of these modules not only significantly improves the model’s processing efficiency but also enhances its adaptability in complex backgrounds. **Figure 3** shows the overall architecture of the improved YOLOv11. With the integration of these new modules, YOLOv11 not only successfully achieves a lightweight design but also maintains segmentation accuracy comparable to the original model, demonstrating its unique advantages particularly in agricultural image segmentation tasks and the specific improvements are as follows:

(1) To strengthen YOLOv11’s capability in detecting and segmenting small-scale targets, this work replaces the default CSPDarknet backbone with EfficientNetV2. This architecture is designed for efficiency and incorporates several enhancements, including optimized structural design, faster training convergence, progressive learning techniques, and adaptive regularization strategies. Leveraging its compound scaling approach, EfficientNetV2 allows simultaneous adjustment of depth, width, and input resolution, effectively balancing performance and computational cost. As a result, the model maintains strong accuracy and throughput even under hardware or deployment constraint (Tan and Le, 2021).

Specifically, EfficientNetV2 adopts the fused-MBConv module in its early stages, which combines the advantages of standard convolution and depthwise separable convolution. It reduces memory use and improves local feature capture using small expansion and 3×3 kernels (Zhang Y. et al., 2024)., and significantly improving the ability to model contour and texture features of small-sized targets such as seedling leaves.Regarding the training paradigm, EfficientNetV2 adopts a progressive learning strategy combined with adaptive regularization techniques. Initially, the model is trained on smaller-sized inputs with mild regularization; as training advances, both image resolution and regularization intensity are incrementally increased. This staged approach helps to manage training complexity effectively. The mechanism has shown improved robustness and generalization performance in detection and segmentation scenarios involving intricate background conditions.

(2) To address the limitations of the original SPPF module—specifically its reliance on fixed-size pooling kernels and its inadequacy in modeling intricate spatial dependencies—we incorporate the Focal Modulation module to enhance the detection of small objects and extraction of fine-grained features in visually complex environments. Grounded in attention mechanisms, this module offers a more adaptive and expressive framework for contextual representation, significantly improving the model’s sensitivity to salient regions within the input image (Yang et al., 2022).Focal Modulation replaces standard attention with a more efficient way to capture context.The module introduces a “focal contextualization” design, which stacks several layers of depth-wise convolution to capture features across multiple spatial scales—allowing the model to understand structural hierarchies from localized patterns to global image context. It also integrates a “gated aggregation mechanism” that selectively merges multi-scale contextual information using learnable gates, amplifying semantically important areas while suppressing extraneous background conten (Liu et al., 2024). The fused context is then injected back into the query path via “element-wise modulation,” enabling position-aware, content-adaptive modulation of feature responses and enhancing the semantic expressiveness of the final outputs.

**Figure 4:**
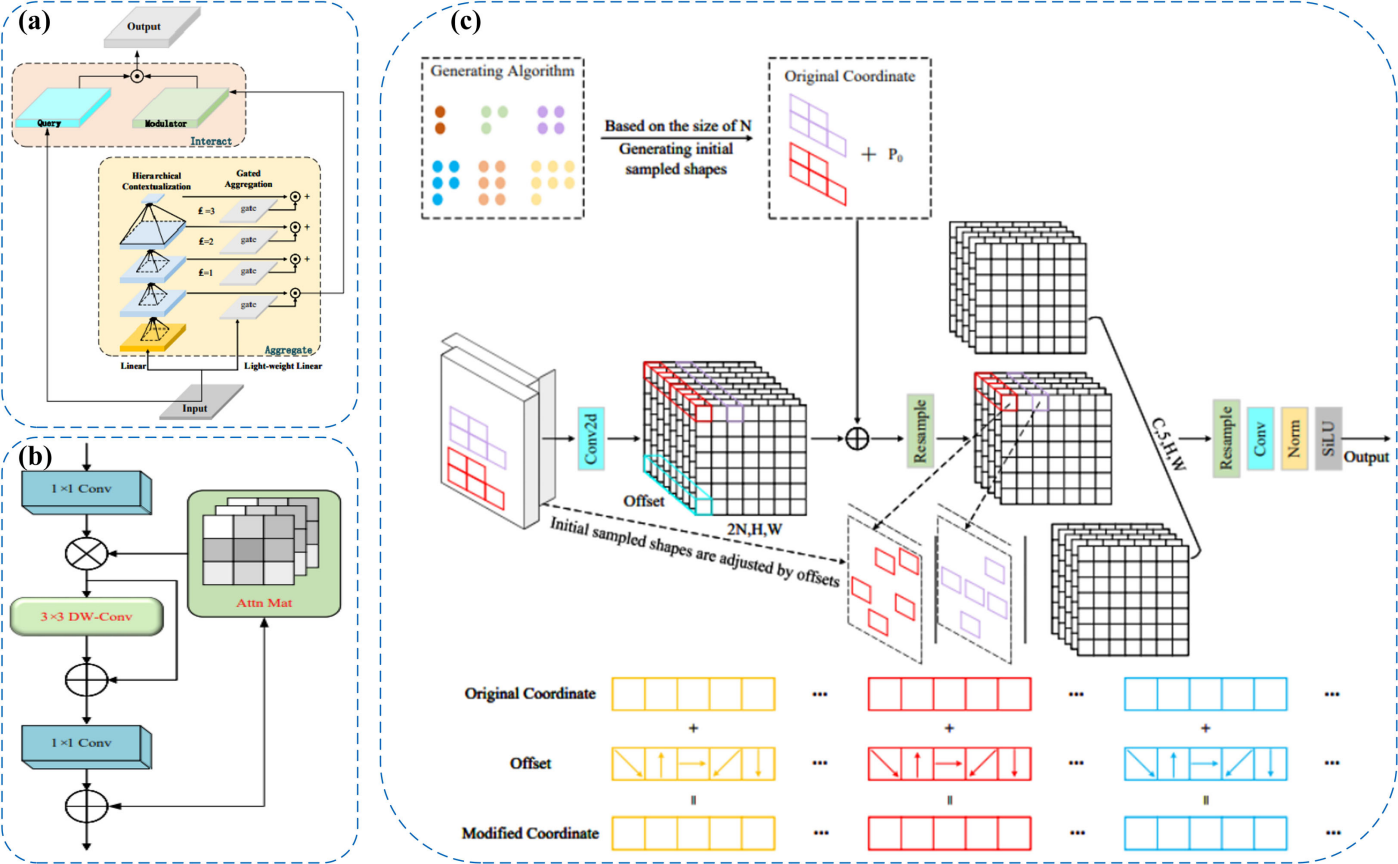
**(a)** Focal Modulation module for enhanced context modeling. **(b)** C2PSA_iRMB module with inverted residual blocks and self-attention. **(c)** LDConv module for efficient shape modeling and edge detection.

As illustrated in **Figure 4a**, Focal Modulation introduces key improvements over conventional self-attention by streamlining the processes of “Query–Key interaction” and “Query–Value aggregation.” By eliminating high-order fully connected operations, it enhances the module’s contextual awareness through spatially adaptive convolution and gated fusion mechanisms. At the initial stage, input features are processed by several convolutional layers to extract progressively scaled contextual information. These features are then aggregated and selectively weighted using a gating mechanism to generate a modulation tensor. This modulator subsequently performs point-wise interactions with the query features, resulting in content-aware feature enhancement. The design effectively decouples contextual encoding from feature modulation, allowing the model to remain lightweight while achieving robust representation capacity for handling intricate image patterns.

(3) The original C2PSA module in YOLOv11 captures cross-level contextual information by embedding self-attention into the CSP structure, but it mainly focuses on spatial information and tends to overlook fine-grained differences between channels. Therefore, we adopted the C2PSA_iRMB module, which integrates the Inverted Residual Mobile Block (iRMB) and the Enhanced Window Multi-Head Self-Attention mechanism (EW-MHSA) in the cross-stage connections, further enhancing the model’s contextual modeling ability and fine-grained feature recognition capability under complex backgrounds, while balancing the efficiency of local information compression and global semantic dependency modeling (Zhang et al., 2023).

The central concept behind the iRMB (Inverted Residual Mobile Block) is to incorporate Transformer-inspired dynamic modeling into compact CNN architectures, enabling efficient processing in dense prediction tasks. Structurally, it extends the design philosophy of the Inverted Residual Block (IRB) by integrating 3×3 depthwise separable convolution (DW-Conv), 1×1 convolutional layers for channel compression and expansion, and attention modules such as ACmix or custom Attn Mat. Specifically, the 1×1 convolutions regulate the dimensionality of feature channels, DW-Conv layers extract spatial positional features, and the attention components enhance global semantic association across disparate regions of the feature map. This design effectively reconciles the needs for localized structural perception and long-range context modeling.Additionally, the iRMB architecture incorporates the Meta-Mobile Block, which achieves structural variability at the module level by combining diverse expansion ratios with learnable operations. This approach enhances the network’s adaptability and generalization across image inputs with varying complexity. As illustrated in **Figure 4b**, the iRMB follows a “bottleneck convolution nested with self-attention” scheme: initially, a 1×1 convolution reduces the channel dimension, followed by a 3×3 depthwise separable convolution to capture spatial characteristics. A lightweight attention mechanism is then applied for global context extraction. Finally, another 1×1 convolution restores the channel depth and establishes a residual connection with the input. This layered configuration improves computational efficiency while strengthening inter-feature interactions, making the design particularly advantageous for complex visual tasks involving high-density small objects and intricate backgrounds.

(4) Traditional convolutional operations are limited in their ability to adapt to spatial variations in object shape (Butkiewicz, 2010). Traditional DCN improves flexibility but becomes costly as kernel size increases (Dong et al., 2025). To address these issues, we propose the LDConv module, which improves the model’s capability to represent irregular object geometries and enhances boundary localization using a more computationally efficient linear offset strategy. Architecturally, LDConv incorporates a coordinate generation mechanism coupled with linear offset computation, allowing the sampling process to adaptively deform while maintaining a controlled parameter budget. This design supports better runtime performance and structural adaptability (Zhang X. et al., 2024). Specifically, LDConv begins by generating a regular set of initial sampling points derived from the kernel size using a coordinate generation procedure. It then applies learnable linear offsets to refine these positions, forming a dynamic sampling grid that conforms to the geometric contours of the target. This allows for more accurate and adaptable convolution operations across localized areas of the input feature map, while ensuring linear scalability in terms of both parameter count and computational burden.

As depicted in **Figure 4c**, LDConv operates through three main stages: generation of base sampling coordinates, prediction of offset values, and convolution-based resampling. Initially, a lightweight convolution is applied to derive offset parameters from the input features. These are combined with the predefined sampling locations to determine the actual sampling points, which are then used to extract feature information via standard convolution. This flexible architecture enables real-time adaptation to diverse object shapes and facilitates multi-scale feature refinement. As a result, it is particularly effective in handling fuzzy contours, densely packed small objects, and structurally intricate regions. The final feature maps, post-normalization and activation, can be directly fed into the main backbone for further processing.

(5) To enhance feature fusion in scenarios involving multi-scale objects, we integrate a lightweight context-aware fusion component—CCFM (Convolutional Context-aware Fusion Module)—into the Neck of YOLOv11, replacing the original FPN (Feature Pyramid Network) and PAN (Path Aggregation Network). Traditional FPN and PAN architectures rely on fixed hierarchies for inter-scale information exchange. Although they enable multi-scale processing to some extent, their static topologies often lead to semantic inconsistencies between shallow and deep layers, and excessive feature aggregation—especially in complex scenes with clutter, occlusion, or dramatic scale variation (Wu et al., 2023). These issues hinder the precise modeling of small objects and edge features.To overcome these limitations, CCFM employs a multi-branch context modeling scheme coupled with a gated fusion strategy, enabling the adaptive weighting of features across scales. Its architecture allows information flow strength to be modulated dynamically based on semantic content during inter-level fusion. This alleviates the fine-detail suppression commonly seen in traditional pyramid networks when facing visually complex environments (Zhao et al., 2024). More specifically, CCFM first encodes features from various scales in a unified manner, applies a context-sensitive gating unit to model the relative importance of each, and produces a single fused output with enhanced discriminative power. While maintaining computational efficiency, this design significantly improves the model’s responsiveness to occlusions, overlaps, and background clutter.

### 2.4 Evaluation metrics for broccoli seedling leaf area features

#### 2.4.1 Model training configuration

In this experiment, the operating system used was Windows 11, with hardware configuration including an Intel Core i9-13900K processor and an NVIDIA GeForce RTX 4090 graphics card. The development environment was Python 3.10.16, with the deep learning framework PyTorch 2.6.0 and CUDA version 12.4. Detailed training parameters of the model are listed in **Table 2**.

**Table 2:** Model training parameters.

Parameters name	Parameters value
Epoch	100
Batch size	16
Image size	640×640
Optimizer	SGD
Learning Rate	0.01
Momentum	0.937
Weight Decay	5×10^-4^

The model’s performance in segmenting the broccoli leaf area was evaluated through instance segmentation analysis. Precision (P), Recall (R), and mean Average Precision (mAP) served as the metrics to assess the model’s accuracy. Meanwhile, model complexity was measured using the number of parameters (Params), floating point operations per second (FLOPs), and weight size (Weight Size).During model training, the input image size was set to 640×640 pixels, and the total number of training iterations was 100. To ensure fairness and comparability of the ablation and comparative experiments, no pre-trained weights were used in any of the experiments.

#### 2.4.2 Model evaluation

We used instance segmentation to evaluate model performance in broccoli leaf area segmentation.This study mainly adopted mean Average Precision (mAP) and model complexity metrics to assess the performance of the proposed model. mAP50 refers to the average precision when the Intersection over Union (IoU) threshold is set to 0.5, which reflects the model’s detection capability under moderate overlap conditions. mAP50–95 is calculated by averaging the AP values under IoU thresholds ranging from 0.5 to 0.95 in steps of 0.05, thus providing a more comprehensive and stringent evaluation of the model’s detection performance.

We adopt two standard metrics to assess computational complexity: FLOPs and Parameters. FLOPs (Floating Point Operations) quantify the total number of arithmetic operations performed during a single forward propagation through the model, while Parameters denote the overall count of trainable weights and biases within the architecture. These indicators jointly reflect the model’s computational load and structural efficiency, and are particularly informative in deployment contexts where hardware resources are constrained. The precise formulas [Equations 1–6] used to compute these metrics are outlined below.


(1) $$P=\frac{TP}{TP+FP}\;$$

(2) $$R=\frac{TP}{TP+FN}$$

(3) $$AP={\int^{}}_{0}^{1}P\left(R\right),dR\;$$

(4) $$mAP=\frac{1}{C}\sum_{i=1}^{C}AP_{i}$$

(5) $$mAP=\frac{1}{C}\sum_{i=1}^{C}AP_{i}$$

(6) $$mAP_{50:95}=\frac{1}{10c}{\sum^{}}_{i=1}^{c}{\sum^{}}_{j=1}^{10}AP_{i}^{IoU=0.5+0.05\left(j-1\right)}$$

Among them, TP, FP, and FN represent true positives, false positives, and false negatives, respectively, and C denotes the total number of categories. To evaluate the structural complexity and lightweight characteristics of the model, this study introduces two computational metrics: Parameters and FLOPs. Parameters refer to the total number of trainable weights and biases in the network, and the calculation formula [Equation 7] is as follows:


(7) $$Params=C_{in}\times K^{2}\times C_{out}$$

Where ${\text{C}}_{\text{in}}$ is the number of input channels, ${\text{C}}_{\text{out}}$ is the number of output channels, and K is the kernel size. FLOPs (Floating Point Operations) refer to the total number of floating point operations required for the model to complete a single forward pass, and are primarily used to evaluate the computational complexity of the model. The calculation formula [Equation 8] is as follows:


(8) $$FLOPs=2\times H\times W\times \left(C_{in}\cdot K^{2}+1\right)\cdot C_{out}$$

Here, H and W denote the height and width of the output feature map, respectively, and the constant term “1” accounts for the bias included in each convolutional kernel.

In summary, the mean Average Precision (mAP) serves as a key metric for evaluating detection accuracy, while FLOPs and Parameters are used to quantify computational demands and memory consumption. Together, these metrics provide a comprehensive view of the model’s practical deployability.

### 2.5 Comparative test

**Figure 5:**
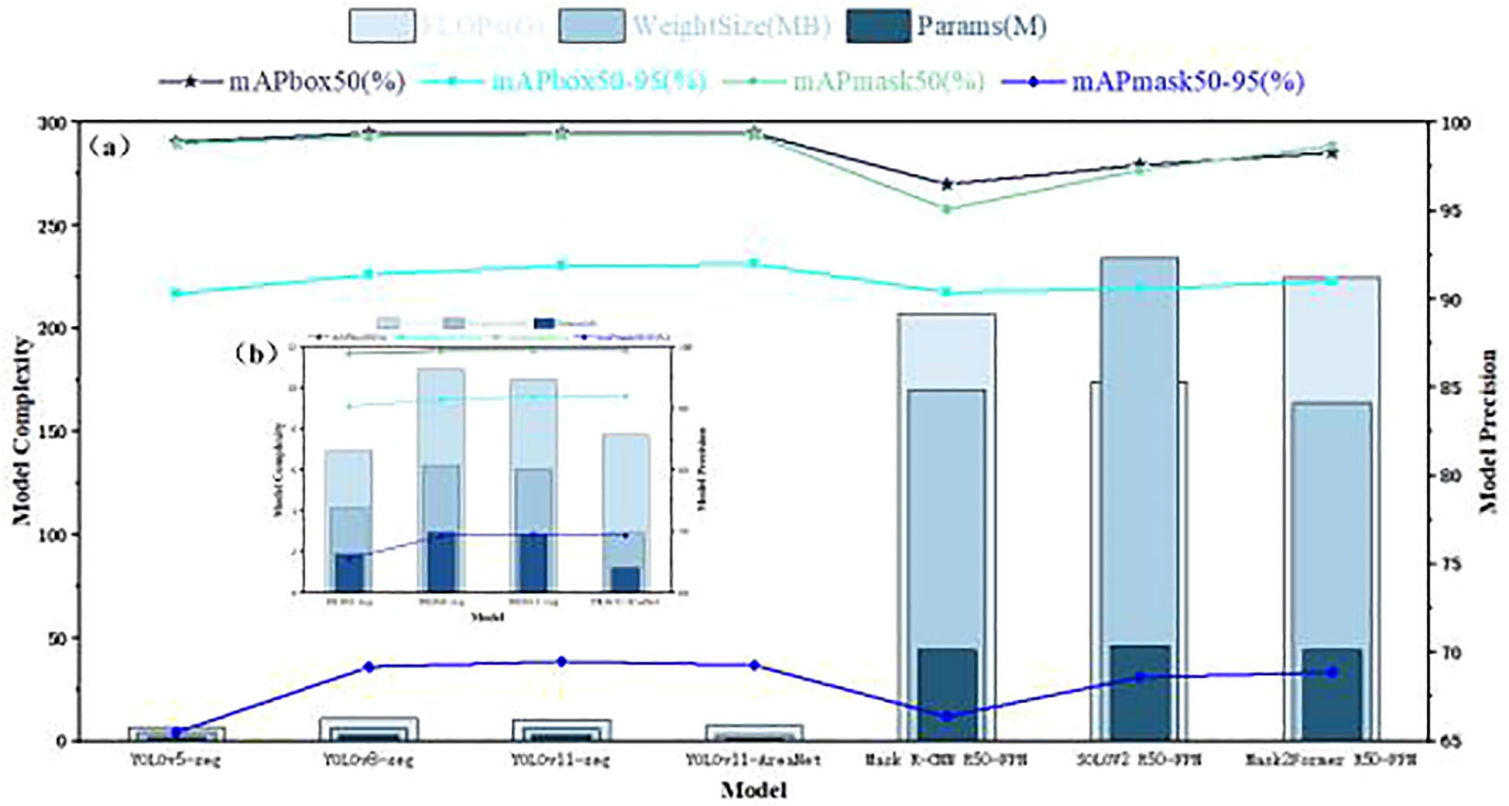
Comprehensive comparison of model performance**.(a)** Overall comparison of segmentation and detection performance among seven models, including YOLO-based and transformer-based methods. **(b)** A zoomed-in view focusing on four lightweight YOLO variants (YOLOv5-seg, YOLOv8-seg, YOLOv11-seg, and YOLOv11-AreaNet) for clearer comparison in model complexity and precision.

To thoroughly assess the instance segmentation capabilities of the proposed YOLOv11-AreaNet, we conducted a series of controlled comparative experiments against several widely used object detection and segmentation frameworks, namely YOLOv5-seg, YOLOv8-seg, and the baseline YOLOv11-seg. In addition, we included three commonly used but computationally heavy instance segmentation models—Mask R-CNN (R50-FPN) (He et al., 2020), SOLOv2 (R50-FPN) (Wang et al., 2020), and Mask2Former (R50-FPN) (Cheng et al., 2022)—to broaden the scope of comparison. All models were trained and tested under the same settings to ensure fairness. As illustrated in **Figure 5a**, the performance of these seven models was analyzed across seven key metrics: parameter count (Params), computational complexity (FLOPs), model file size (Weight Size), detection accuracy (mAPbox50, mAPbox50–95), and segmentation accuracy (mAPmask50, mAPmask50–95). Despite their use in many segmentation tasks, Mask R-CNN, SOLOv2, and Mask2Former show no clear advantage in accuracy while exhibiting extremely large model sizes and computational burdens.

To better highlight the performance of lightweight models, we additionally present **Figure 5b**, which focuses solely on YOLOv5-seg, YOLOv8-seg, YOLOv11-seg, and the YOLOv11-AreaNet. This zoomed-in comparison provides a clearer view of the trade-off between accuracy and model efficiency within the YOLO family.

According to the experimental results, YOLOv11-AreaNet shows comparable or even slightly improved accuracy compared to YOLOv11-seg (with mAPbox50–95 increased to 92.0% and mAPmask50–95 reaching 69.3%), while its number of parameters is reduced from 2.84M in the original model to 1.21M, FLOPs decrease from 10.4G to 7.7G, and the model weight size is significantly compressed to only 2.9MB, representing reductions of 57.4%, 25.9%, and 51.7%, respectively. This shows the model uses fewer resources without losing accuracy, making it easier to deploy. Although YOLOv5-seg and YOLOv8-seg exhibit certain advantages in segmentation accuracy, their models are large and inference efficiency is low. In particular, YOLOv8-seg has FLOPs reaching 10.9G and model weight size up to 6.2MB, posing certain challenges for deployment on edge devices. In contrast, YOLOv11-AreaNet achieves comparable accuracy to YOLOv8-seg while significantly compressing model complexity, showing stronger lightweight capability and deployment flexibility.

In summary, YOLOv11-AreaNet demonstrates a well-optimized trade-off between precision and computational efficiency, rendering it particularly suitable for deployment on agricultural terminal devices with limited resources. Its strong engineering applicability and deployment readiness make it a compelling solution for instance segmentation tasks involving broccoli seedling leaves.

### 2.6 Ablation test

**Figure 6:**
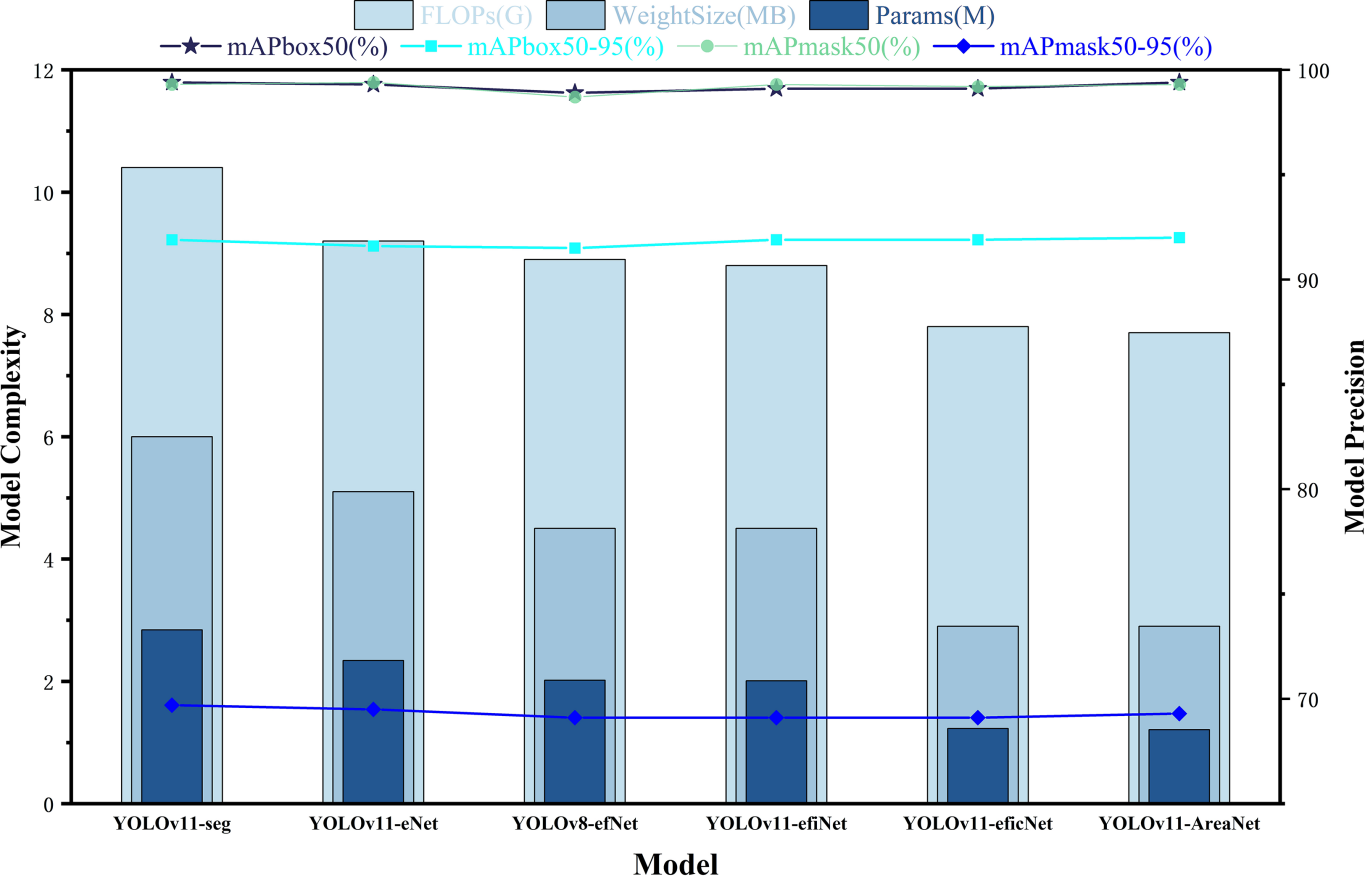
Ablation study results of the model.

To assess the individual contributions of each proposed component to overall model performance, we conducted a structured ablation study. Starting with the original YOLOv11-seg as the baseline, we incrementally incorporated the EfficientNetV2 backbone, the Focal Modulation module for contextual awareness, the iRMB lightweight attention mechanism, the CCFM structure for cross-scale fusion, and the LDConv deformable convolution module—ultimately assembling the complete YOLOv11-AreaNet architecture. In each experimental iteration, only one architectural modification was applied, while all training configurations and hyperparameters were held constant to ensure fair and valid comparisons. The results, including segmentation accuracy, parameter count, computational complexity (FLOPs), and model weight size, are visualized in **Figure 6**.

According to the experimental results, in the first stage, after introducing EfficientNetV2 (YOLOv11-eNet), the number of parameters decreased from 2.84M to 2.34M, FLOPs dropped to 9.2G, and the model weight size was reduced to 5.1MB, while the accuracy remained stable, with mAPbox50–95 and mAPmask50–95 reaching 91.6% and 69.5%, respectively. Further replacing the original backbone with a compound scaling structure (YOLOv8-efNet and YOLOv11-efiNet) reduced the parameters and FLOPs to 2.01M and 8.8G, respectively, while detection and segmentation accuracy remained stable at 91.9% and 69.1%, indicating that the introduction of EfficientNetV2 effectively compressed the model weight size without affecting accuracy.Subsequently, after adding the Focal Modulation and iRMB modules (YOLOv11-eficNet), the computational cost further decreased to 7.8G, the parameters were compressed to 1.23M, and the weight size was only 2.9MB. The model still maintained 91.9% mAPbox50–95 and 69.1% mAPmask50-95, indicating that Focal Modulation and iRMB can enhance contextual modeling and local attention capabilities under low computational cost, improving the model’s recognition ability for small targets and complex backgrounds.Finally, the fully constructed YOLOv11-AreaNet integrated all five structural improvements, with the number of parameters further reduced to 1.21M (approximately 55.4% reduction), FLOPs reduced to 7.7G (approximately 48.6% reduction), and the model weight size compressed to 2.9MB (approximately 54.7% reduction). Meanwhile, mAPbox50–95 and mAPmask50–95 increased to 92.0% and 69.3%, respectively, achieving the dual objective of “lightweight design” and “high precision.”

In addition to numerical comparisons, we further analyzed the interaction and effectiveness of each module. We found that EfficientNetV2 provides a strong foundation by reducing model size while maintaining accuracy, which is further enhanced by Focal Modulation’s ability to capture contextual dependencies. When used together, these two modules exhibit a synergistic effect, resulting in greater gains than using either module individually. However, as more modules such as iRMB, LDConv, and CCFM are added, the performance improvement tends to plateau, indicating a trend of diminishing returns. Moreover, we observed that certain modules show stronger advantages under specific conditions: LDConv contributes more under scenarios with overlapping leaves due to its deformable edge perception, while Focal Modulation is more effective under complex illumination or cluttered backgrounds. These observations provide deeper insight into how each module contributes not only individually but also collectively to the overall segmentation performance.

## 3 Segmentation results and leaf area estimation

### 3.1 Visualization-Based comparative analysis

**Figure 7:**
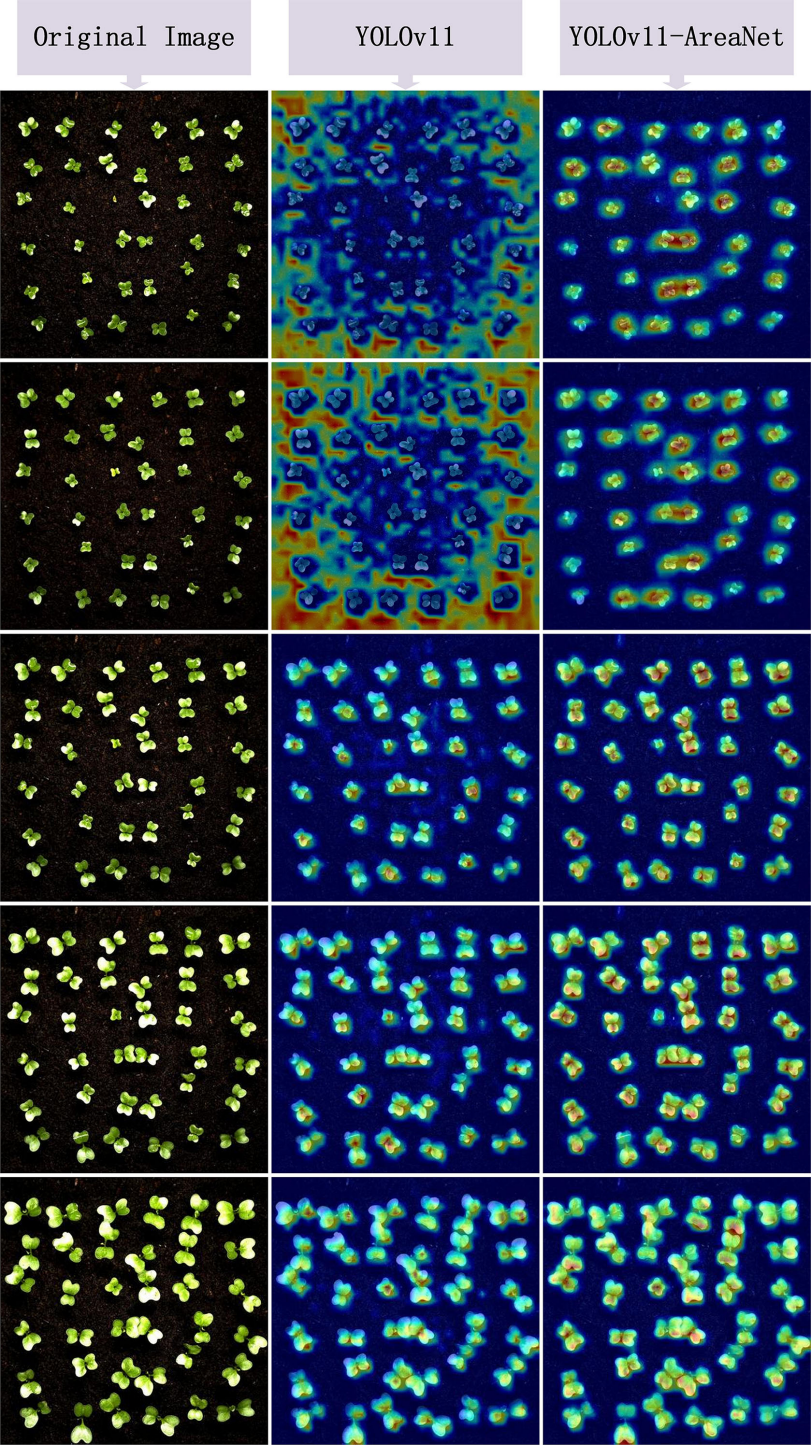
Eigen CAM heatmap comparison results.

In order to further evaluate the effectiveness and focus capability of the improved model in leaf instance segmentation, we utilized the EigenCAM technique to visualize the model’s output. Through the heatmaps, we can intuitively observe the model’s response intensity to the target regions and thereby determine whether its attention distribution is more reasonable. **Figure 7** shows the visualization results of the heatmap comparison. Each group displays three columns of images: the original image, the heatmap of the YOLOv11 model, and the heatmap of the improved YOLOv11-AreaNet model.

From the comparison results of the heatmaps, it can be observed that the original model exhibits problems such as scattered activation regions, blurred edges, and insufficient attention to small leaves in many samples. Some areas even show misactivation or missed detection of targets. In contrast, YOLOv11-AreaNet presents more focused and reasonably covered response regions in most images. Especially at the edges and overlapping regions of broccoli seedling leaves, the CAM response is more concentrated and the object boundaries are clearer, which effectively improves the model’s recognition robustness under complex backgrounds. This phenomenon indicates that the introduced Focal Modulation, iRMB attention mechanism, and LDConv edge modeling capability enhance the model’s ability to capture local features and perceive contextual information, making the model structurally more sensitive to targets and more complete in representation.

**Figure 8:**
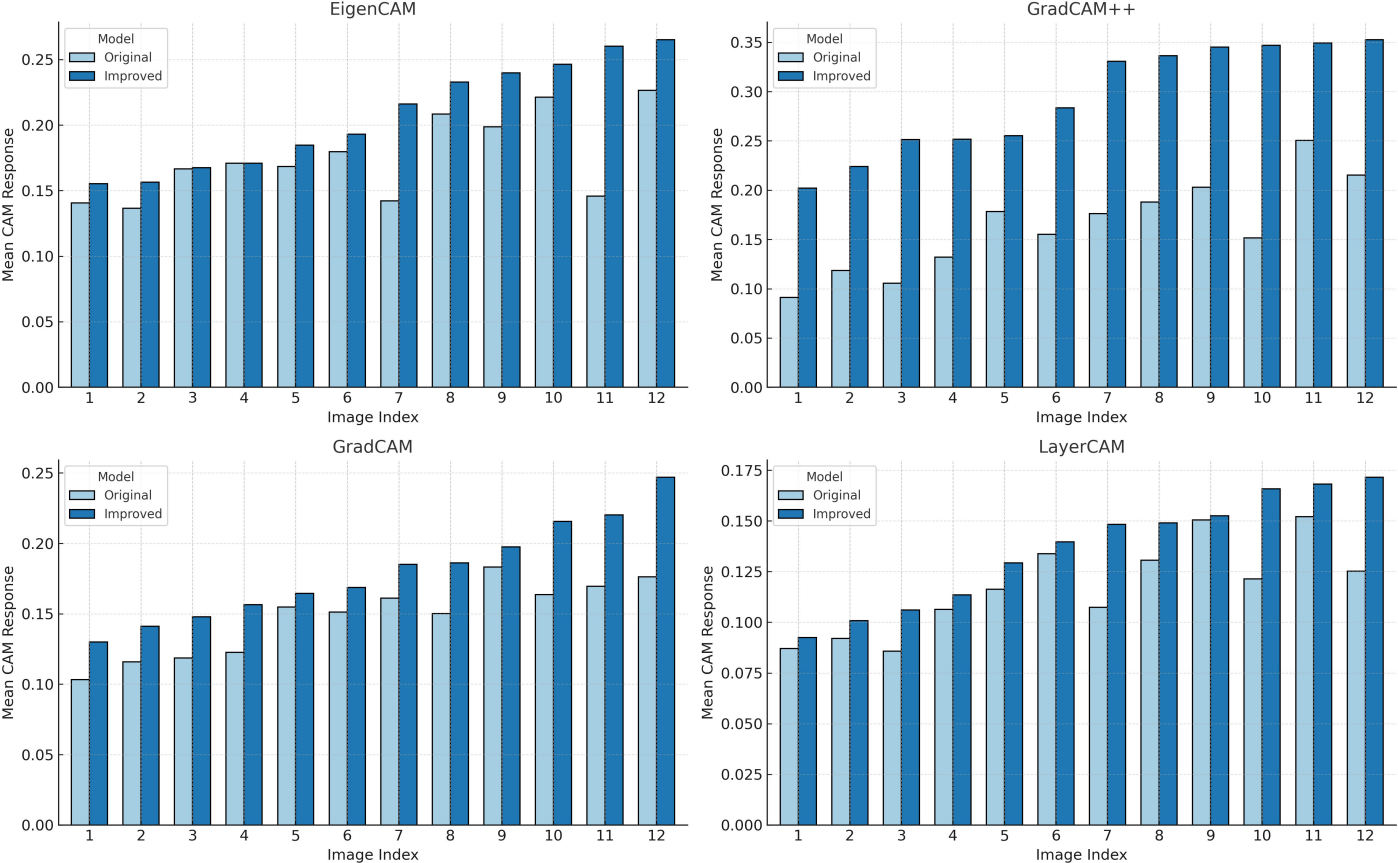
Comparison of activation responses across four CAM methods.

In addition, to quantify the differences in activation responses between different models, we calculated the average activation values across 12 images under four CAM methods and plotted bar charts as shown in **Figure 8**. According to the statistical results, regardless of whether Grad CAM, Grad CAM++, Layer CAM, or Eigen CAM was used, YOLOv11-AreaNet had stronger activation than the original model, especially in Grad-CAM++ and EigenCAM.This indicates that in the segmentation task, the improved model not only covers the key target regions more effectively but also differentiates the target from the background with greater accuracy, thereby enhancing the overall completeness of the semantic understanding.

Based on the comprehensive visualization analysis, it is clear that, through structural optimization, YOLOv11-AreaNet not only achieves a lightweight design but also significantly enhances the model’s focus on complex structures and fine-grained leaves, as well as its spatial resolution capability. This further supports the effectiveness and interpretability of the proposed improvements.

### 3.2 Post-processing of leaf segmentation results

#### 3.2.1 Leaf area segmentation processing

To accurately extract valid contours from the segmentation masks of broccoli leaves output by the model and to calculate the leaf area, a series of image processing techniques were introduced in this study as post-processing steps based on the segmentation results. These include morphological operations, binarization, edge detection, region segmentation, geometric measurement, and visual mapping. The specific methods and formulas are described as follows:

(1)To eliminate segmentation noise and edge discontinuities, this study adopts morphological opening and closing operations (Sun et al., 2007). Let the binary image be A and the structuring element be B, then the definitions of morphological opening and closing operations are given in (Equations 9-12):

Opening (erosion followed by dilation) is defined as:


(9) A∘B=(A⊖B)⊕B

Closing (dilation followed by erosion) is defined as:


(10) A·B=(A⊕B)⊖B

Where the erosion and dilation operations are defined as follows:


(11) $$\left(A⊖B\right)\left(x,y\right)=\underset{\left(u,v\right)\in B}{min}A\left(x+u,y+v\right)$$

(12) $$\left(A⊕B\right)\left(x,y\right)=\underset{\left(u,v\right)\in B}{max}A\left(x+u,y+v\right)$$

where: ⊖ denotes the erosion operation ⊕ denotes the dilation operation

These operations help smooth object contours, eliminate small artifacts, and bridge narrow gaps in the segmented mask.

(2) To achieve automatic binarization of images, the Otsu adaptive thresholding method is introduced. Its objective is to maximize the between-class variance σ_B²(t), calculated as shown in Equations 13-16 (Goh et al., 2018):


(13) $$\sigma_{B}^{2}\left(t\right)=w_{0}\left(t\right)w_{1}\left(t\right){\left(\mu_{0}\left(t\right)-\mu_{1}\left(t\right)\right)}^{2}$$

Where the class weights and means are defined as follows:


(14) $$w_{0}\left(t\right)=\sum_{i=0}^{t}p\left(i\right),w_{1}\left(t\right)=\sum_{i=t+1}^{L}p\left(i\right)$$

(15) $$\mu_{0}\left(t\right)=\frac{\sum_{i=0}^{t}ip\left(i\right)}{w_{0}\left(t\right)},\mu_{1}\left(t\right)=\frac{\sum_{i=t+1}^{L}ip\left(i\right)}{w_{1}\left(t\right)}$$

The ultimate goal is to find the optimal threshold:


(16) $$t^{*}=\arg\underset{t}{max}\sigma_{B}^{2}\left(t\right)$$

(3) To extract the leaf contour edges, the Canny algorithm is adopted, which includes gradient calculation, non-maximum suppression, and double threshold connection (Ding and Goshtasby, 2001). The gradient is defined as shown in (Equations 17-18):


(17) $$G_{x}\left(x,y\right)=\frac{\partial I\left(x,y\right)}{\partial x},G_{y}\left(x,y\right)=\frac{\partial I\left(x,y\right)}{\partial y}$$

(18) $$\;\;\;\;G\left(x,y\right)=\sqrt{G_{x}^{2}\left(x,y\right)+G_{y}^{2}\left(x,y\right)},\theta \left(x,y\right)=arctan2\left(G_{y}\left(x,y\right),G_{x}\left(x,y\right)\right)$$

(4) To further refine the segmentation of overlapping leaf regions, a distance-transform-based watershed algorithm is introduced (Tang and Wang, 2006). Its core steps include distance transform and watershed segmentation as defined in (Equations 19-20):

Distance Transform:


(19) $$D\left(x,y\right)=\underset{\left(u,v\right)\in B}{min}\sqrt{{\left(x-u\right)}^{2}+{\left(y-v\right)}^{2}}$$

Watershed Marking and Segmentation:


(20) M(x,y)=watershed(D(x,y))

(5) Based on the extracted contours, the pixel-level leaf area and perimeter are as shown in (Equations 21-25):

Pixel Area:


(21) $$A={\int^{}}_{\Omega }1dxdy\;$$

Pixel Perimeter:


(22) $$P={\int^{}}_{\partial \Omega }1ds\;$$

By applying a pixel-to-physical unit conversion factor, the actual area and perimeter can be obtained:


(23) $$A_{real}=A_{pixels}\times \Delta x\times \Delta y,P_{real}=P_{pixels}\times \Delta x$$

To ensure that the segmentation results carry a rigorous physical interpretation during the calculation of leaf area and perimeter, all images in this study were uniformly cropped to a resolution of 1500 × 1500 pixels. This region corresponds precisely to the full field of view of the cultivation tray, covering an area of 25 cm × 25 cm. Based on this, the pixel-to-centimeter conversion factors Δx and Δy were defined to represent the actual physical length corresponding to a single pixel in the horizontal and vertical directions, respectively. The formulas are as follows:


(24) $$\Delta x=\frac{L_{x}}{w},\Delta y=\frac{L_{y}}{h}$$

Where $L_{x}$ = $L_{y}$ = 25cm, w = h = 1500pixels。Substituting the values yields:


(25) $$\Delta x=\Delta y=\frac{25}{1500}=\frac{0.0167cm}{pixel}$$

This conversion factor was applied to the pixel-based contour area to obtain results in cm², and similarly, to convert the perimeter, it was multiplied by Δx to yield measurements in cm. This approach not only improves the physical interpretability of the leaf area measurements but also ensures the consistency and comparability of the quantitative results across different samples.

#### 3.2.2 Visualization of the image processing pipeline and transformation path analysis

**Figure 9:**
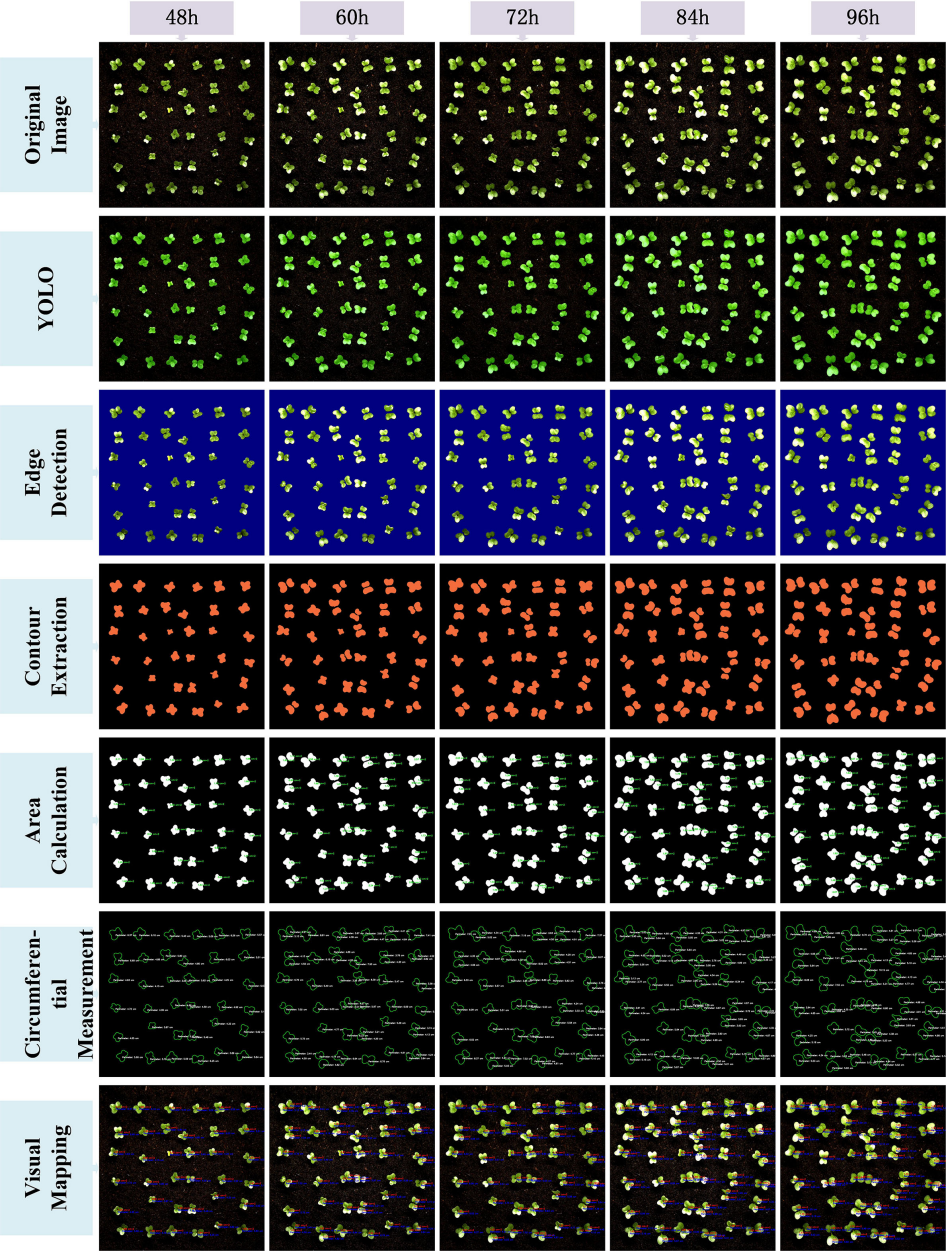
Visualization of the image processing workflow for different time points (48h to 96h).

**Figure 10:**
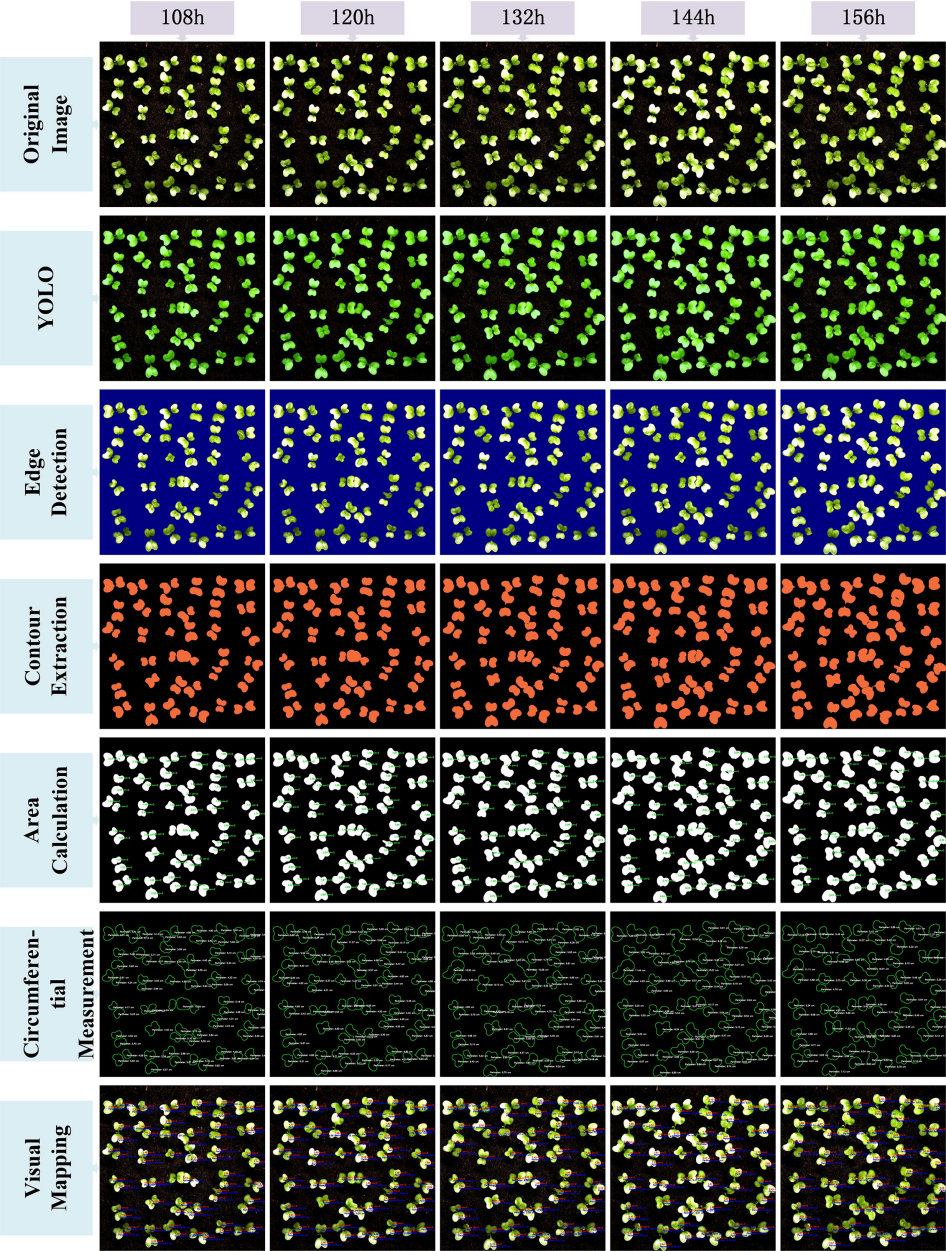
Visualization of the image processing workflow for different time points(108h to 156h).

After completing the above image processing steps, the results were visualized to demonstrate the full conversion process from the YOLO model-predicted segmentation masks to the final area extraction. **Figures 9** and **10** illustrate the seven key steps involved, covering multiple time points (from 48 hours to 156 hours), with the aim of clearly showing the progression from coarse predictions to precise geometric measurements.To enhance the interpretability of the post-processing workflow, each row in **Figures 9** and **10** corresponds to a specific step in the image analysis pipeline. The first row shows the original image, serving as the baseline reference. The second row presents the YOLO-based segmentation result, highlighting detected leaf regions. The third row applies Canny edge detection to emphasize leaf boundaries. In the fourth row, contour extraction is performed to isolate leaf outlines. The fifth and sixth rows display the area and perimeter calculations, where pixel-wise masks are analyzed to quantify morphological characteristics. Finally, the seventh row illustrates the visual mapping, using pseudocolor overlays to provide intuitive feedback on leaf size and shape. This step-by-step structure reflects the complete transformation from raw input to quantitative output and facilitates transparent understanding of the analysis pipeline.

The original image is first processed by the YOLOv11-AreaNet model to generate an initial leaf segmentation mask. Morphological operations are then applied to refine the mask by removing noise and smoothing the edges, leading to more precise delineation of the leaf regions. To further improve the segmentation results, the optimized mask is overlaid onto the original image using a semi-transparent technique, ensuring the segmented areas align more closely with the details of the original image.

Background removal is then applied to isolate the pure leaf regions, preparing the image for the next stage of feature extraction. During this process, edge detection is employed to capture the detailed contours of the leaves, ensuring precise localization of the boundaries. Following this, the leaf area is calculated from the binarized image, guaranteeing the accuracy of the computed results.The edge map is then combined with the contour map to further refine the leaf boundary and calculate the perimeter. This series of processing steps effectively illustrates the progression from rough segmentation to precise geometric measurement, providing reliable data for leaf geometric feature analysis and subsequent applications.Finally, a visual mapping image is created, overlaying the refined contours along with area and perimeter information onto the original image, facilitating further analysis and presentation. These steps not only improve the segmentation performance of the YOLO model but also significantly enhance the accuracy and visualization quality of leaf segmentation through image processing techniques, providing strong support for subsequent automated leaf analysis.

From the image visualization, it can be observed that the morphological operations effectively eliminate boundary breakages and noise spots in the segmentation results, enabling more stable edge detection in the subsequent steps. The leaf contour boundaries extracted by the Canny algorithm exhibit good continuity and closure, which facilitates the watershed algorithm in effectively segmenting overlapping leaf regions. Meanwhile, the final area calculation results are visualized to provide an intuitive perception of leaf contours and area size.

This image processing workflow not only effectively reflects the underlying algorithmic logic but also generates verifiable intermediate outputs that validate the accuracy of the subsequent leaf area measurements. The post-processing pipeline established in this study provides strong visual interpretability and demonstrates robust capability in separating complex targets, such as occluded, overlapping, or morphologically irregular plant leaves.In comparison to direct area estimation from segmentation masks, this workflow effectively minimizes cumulative errors and structural ambiguity, ensuring that the final area computation results are stable and highly reproducible.

#### 3.2.3 Comparative analysis with manual leaf area measurements

**Figure 11:**
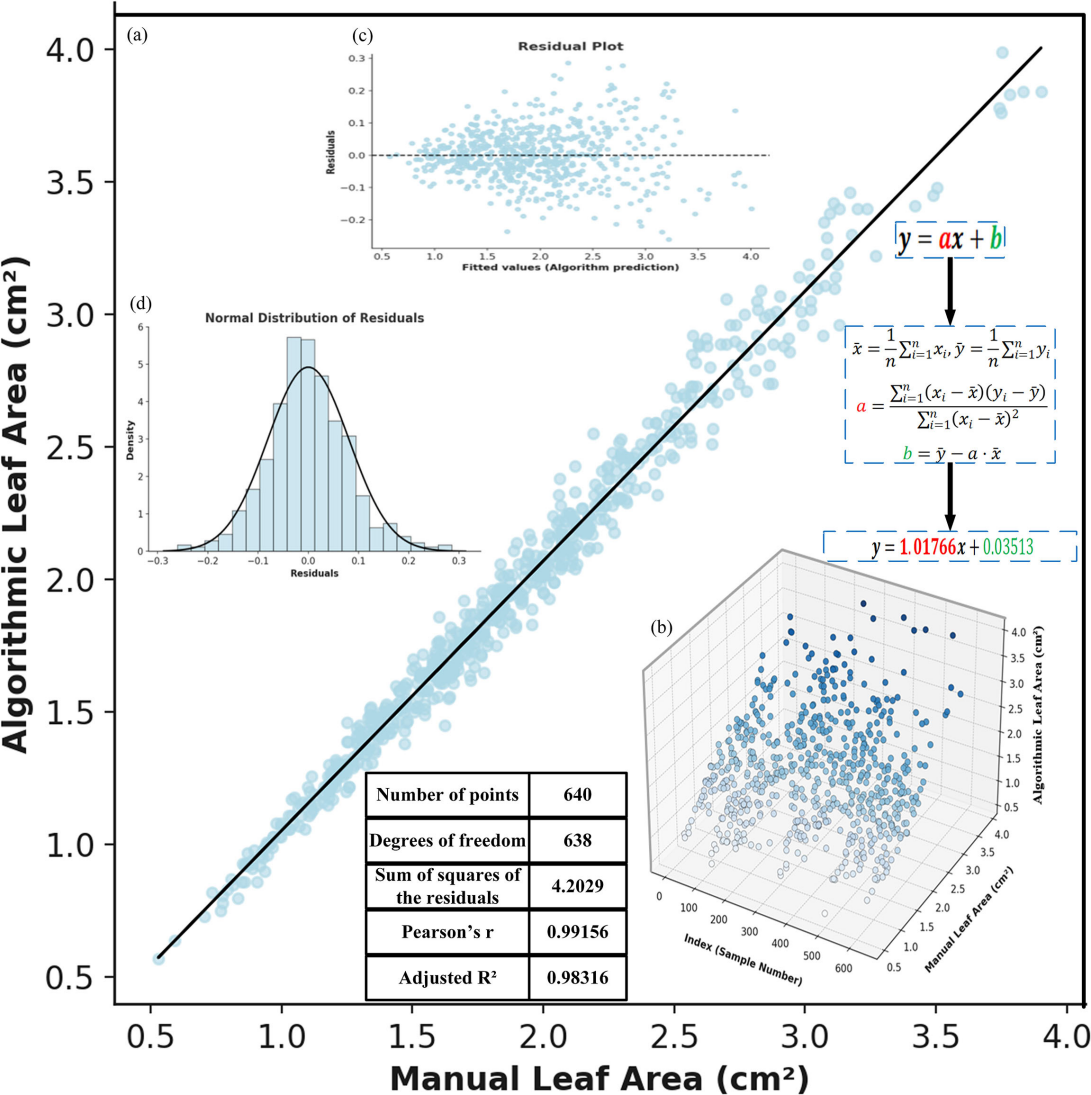
Correlation analysis between manual and algorithmic leaf area measurements: **(a)** fitted straight line. **(b)** 3D scatter plot of manual and algorithmic measurements. **(c)** residual plot. **(d)** normal distribution of residuals.

We further verified our method by comparing model-estimated leaf areas with manual measurements.A subset of images was randomly selected from the test set, and leaf area was measured using both manual methods and the automatic extraction process of the model. Based on the obtained data, regression fitting plots, residual plots, and normal distribution plots were constructed (as shown in **Figure 11**) to evaluate the correlation, consistency, and error characteristics between the two measurement methods.

**Figure 11a** illustrates the linear fitting relationship between the manually measured leaf area (x-axis) and the model-predicted area (y-axis). The fitted curve approximates the diagonal line, indicating a significant linear correlation between the two. Based on least squares calculation, the slope of the fitted line is close to 1, and the intercept is close to 0, with a coefficient of determination R² reaching 0.983, demonstrating a high degree of consistency between the model predictions and the ground truth, and verifying the high reliability of YOLOv11-AreaNet in the leaf area estimation task.

**Figure 11b** presents a 3D spatial distribution plot that shows the distribution of different samples (i.e., the measured leaf values in the images) based on both model-based and manual measurements. From the figure, it is evident that the model and manual measurements exhibit a strong linear correlation in the sample space, further validating the consistency between the two measurement methods.

**Figure 11c** displays a residual plot, which illustrates the distribution of deviations between the model predictions and the manually measured values. It can be seen that the majority of residual points are clustered around zero, with no apparent systematic trend, indicating that the errors are minimal and random. This further supports the accuracy and reliability of the fitted model.

**Figure 11d** shows the results of a normality test for the error distribution, illustrating the frequency distribution curve of the measurement errors. The figure suggests that the error distribution closely follows a standard normal distribution, with well-maintained kurtosis and symmetry. This indicates that the errors are caused by random fluctuations rather than any systematic bias in the model, further confirming the scientific validity and stability of the automatic segmentation and area calculation process.

#### 3.2.4 Single-leaf tracking and dynamic leaf area analysis

**Figure 12:**
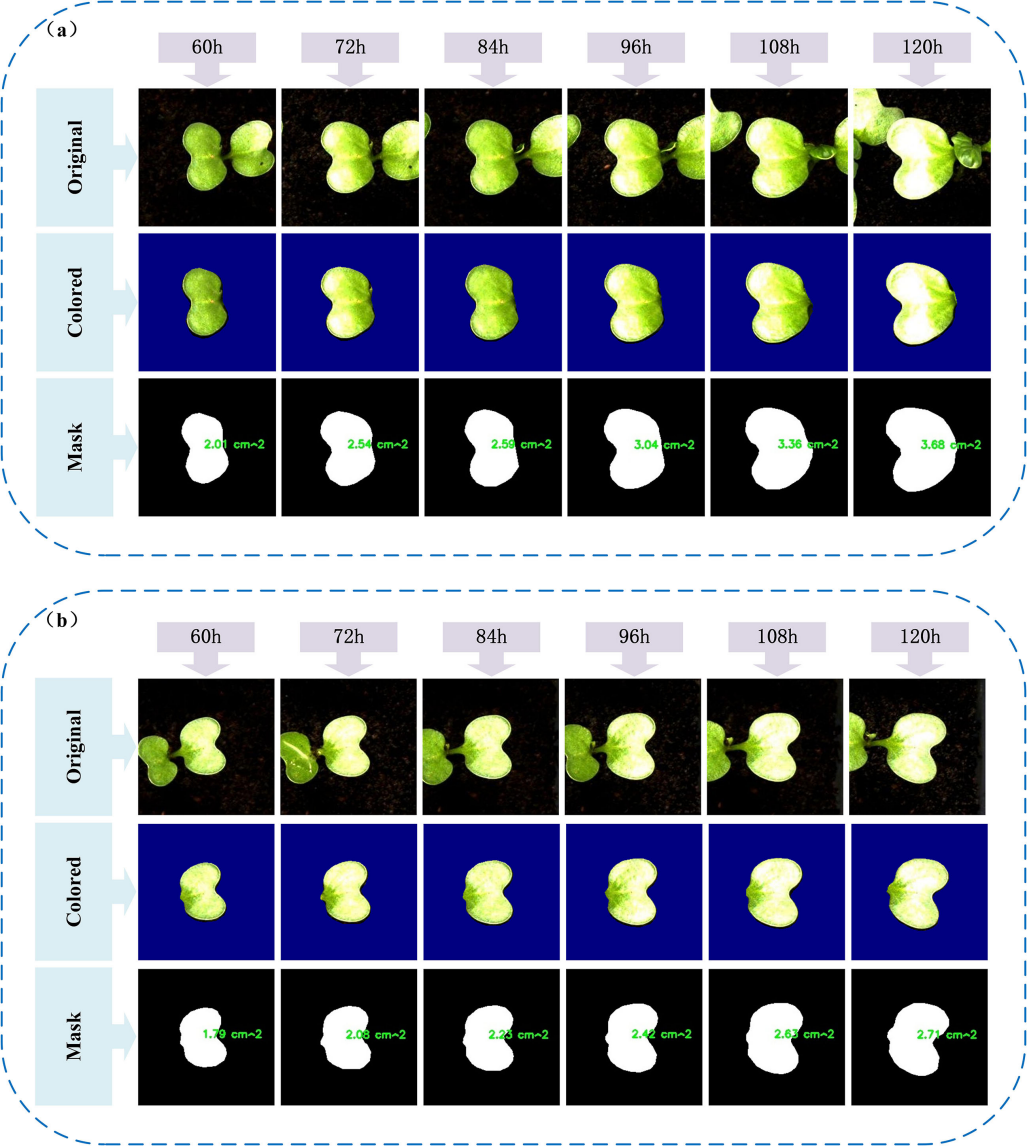
Visualization of typical leaf growth: **(a, b)** Original images, segmented masks, and calculated leaf areas of two representative leaves from 60h to 120h.

**Figure 13:**
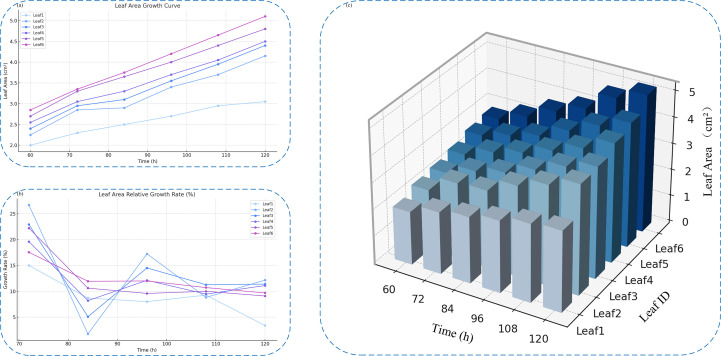
Analysis of individual leaf growth over time. **(a)** Leaf area growth curves of six selected leaves. **(b)** Relative growth rate curves across different time points. **(c)** 3D bar chart showing time-series leaf area variations.

A deeper understanding of the early-stage growth dynamics of individual broccoli seed leaves during germination was gained by selecting representative leaf samples in this study, with their original images, mask-cropped results, and area-annotated images sequentially presented, as shown in **Figure 12**. This sequence clearly illustrates the complete process, from image acquisition to quantitative analysis.On this basis, six representative leaves were further selected to construct a time-series area growth curve (**Figure 13a**), a relative growth rate per unit time plot (**Figure 13b**), and a 3D bar chart (**Figure 13c**), in order to systematically analyze the dynamic growth characteristics of the leaves between 60 and 120 hours.

Analysis of the leaf area growth curves between 60 and 120 hours indicates that all tracked leaves followed a general upward trajectory. However, growth dynamics varied based on initial leaf size. Smaller leaves, such as Leaf1 and Leaf2, exhibited steadier and slower expansion, while larger leaves like Leaf5 and Leaf6 experienced accelerated growth during the later phases—highlighting variability in inherent growth potential.Examination of the relative growth rate per time unit further revealed that leaves such as Leaf2 and Leaf3 showed sharp early-stage increases, with peak rates reaching 26.6%. Yet, these leaves also displayed mid-to-late stage fluctuations, potentially influenced by environmental or physiological factors including light availability or nutrient uptake. In contrast, Leaf4 through Leaf6 demonstrated more consistent growth, remaining within a stable range of 10% to 13%, indicative of a steady developmental rhythm.

The 3D bar visualization clearly maps the interplay between time, leaf identity, and area expansion. This chart illustrates both absolute area disparities at fixed time points and a coordinated overall growth trend. Despite observable individual variability, the collective behavior of the leaves suggests that environmental conditions during the experiment were well-controlled and stable.

## 4 Conclusion

To enable precise segmentation and automated leaf area estimation for small-seeded crops—while addressing the inefficiencies, inaccuracies, and inconsistencies associated with traditional manual approaches—this study centers on broccoli seedlings and introduces YOLOv11-AreaNet, an enhanced instance segmentation framework. Built upon the original YOLOv11, the model incorporates several architectural improvements: EfficientNetV2 serves as the backbone to balance parameter reduction with representational strength; Focal Modulation enhances contextual feature modeling; the lightweight C2PSA-iRMB module strengthens both spatial and channel-wise attention; CCFM is employed in the neck to fuse multi-scale features; and LDConv is integrated to optimize downsampling with deformable perception. Collectively, these refinements lead to substantial compression—achieving reductions of 57.4% in parameters, 25.8% in FLOPs, and 51.7% in model weight size—without compromising segmentation accuracy, thereby offering a lightweight yet high-performing solution with practical deployment potential.

Building on the segmentation of broccoli seedling leaves, the study also establishes a comprehensive post-processing pipeline that bridges model outputs with quantifiable area calculations. This pipeline encompasses mask refinement, edge detection, contour tracing, watershed segmentation, and geometric computation—completing the full workflow from prediction to physical measurement. To assess the alignment between automated and manual leaf area estimates, a multi-faceted statistical validation framework was employed. This included linear regression visualization, residual analysis, swarm distribution plots, and normality testing. Results revealed a high level of agreement between both approaches, with a regression coefficient reaching 0.987 and error distributions conforming to normality without systematic deviation—demonstrating both the reliability and accuracy of the method in real-world agricultural scenarios.

Moreover, leveraging time-series imagery, the study conducted individualized leaf tracking to investigate growth dynamics over time. Six representative leaves were selected for analysis, with visualizations of their area expansion from 60 to 120 hours, per-time-unit growth rates, and corresponding 3D growth surfaces. While all leaves displayed a general upward growth trajectory, clear inter-individual variability was observed: some maintained steady and continuous expansion, whereas others showed irregular fluctuations or noticeable deceleration during the later stages. The 3D bar graphs and rate curves provided an intuitive representation of differences in growth efficiency, revealing a distinct two-phase pattern characterized by “early rapid enlargement followed by gradual slowing.” These findings offer both theoretical insights and empirical data to support plant-level growth modeling and enable fine-scale temporal monitoring.

This work introduces an effective, automated, and precise approach for high-throughput monitoring of leaf area in small-seeded crops, with broccoli serving as the representative case. The proposed method demonstrates strong generalizability and operational feasibility, particularly for large-scale phenotyping applications on resource-limited platforms. Nonetheless, certain challenges persist: segmentation accuracy can be affected by issues like occlusion and leaf adhesion, and the current post-processing workflow still requires enhancements in robustness. Future research will focus on refining structural compression techniques, incorporating multimodal sensing strategies, and extending the applicability of this framework to broader smart agriculture domains—including morphological tracking, disease detection, and temporal growth analysis across diverse crop species. These efforts are expected to support the advancement of digital phenotyping and contribute to modern agricultural innovation.

## Data Availability

The raw data supporting the conclusions of this article will be made available by the authors, without undue reservation.
